# The Effect of Diaphragmatic Breathing on Attention, Negative Affect and Stress in Healthy Adults

**DOI:** 10.3389/fpsyg.2017.00874

**Published:** 2017-06-06

**Authors:** Xiao Ma, Zi-Qi Yue, Zhu-Qing Gong, Hong Zhang, Nai-Yue Duan, Yu-Tong Shi, Gao-Xia Wei, You-Fa Li

**Affiliations:** ^1^Faculty of Psychology, Beijing Normal UniversityBeijing, China; ^2^Collaborative Innovation Center of Assessment toward Basic Education Quality, Beijing Normal UniversityBeijing, China; ^3^College of P.E. and Sports, Beijing Normal UniversityBeijing, China; ^4^Department of Psychology, University of Chinese Academy of SciencesBeijing, China; ^5^Key Laboratory of Behavioral Science, Institute of Psychology, Chinese Academy of SciencesBeijing, China; ^6^Department of Psychiatry, Massachusetts General Hospital, Harvard Medical School, BostonMA, United States

**Keywords:** breathing technique, mental health, real-time feedback, relaxation, sustained attention

## Abstract

A growing number of empirical studies have revealed that diaphragmatic breathing may trigger body relaxation responses and benefit both physical and mental health. However, the specific benefits of diaphragmatic breathing on mental health remain largely unknown. The present study aimed to investigate the effect of diaphragmatic breathing on cognition, affect, and cortisol responses to stress. Forty participants were randomly assigned to either a breathing intervention group (BIG) or a control group (CG). The BIG received intensive training for 20 sessions, implemented over 8 weeks, employing a real-time feedback device, and an average respiratory rate of 4 breaths/min, while the CG did not receive this treatment. All participants completed pre- and post-tests of sustained attention and affect. Additionally, pre-test and post-test salivary cortisol concentrations were determined in both groups. The findings suggested that the BIG showed a significant decrease in negative affect after intervention, compared to baseline. In the diaphragmatic breathing condition, there was a significant interaction effect of group by time on sustained attention, whereby the BIG showed significantly increased sustained attention after training, compared to baseline. There was a significant interaction effect of group and time in the diaphragmatic breathing condition on cortisol levels, whereby the BIG had a significantly lower cortisol level after training, while the CG showed no significant change in cortisol levels. In conclusion, diaphragmatic breathing could improve sustained attention, affect, and cortisol levels. This study provided evidence demonstrating the effect of diaphragmatic breathing, a mind-body practice, on mental function, from a health psychology approach, which has important implications for health promotion in healthy individuals.

## Introduction

Breathing practice, also known as “diaphragmatic breathing” or “deep breathing,” is defined as an efficient integrative body–mind training for dealing with stress and psychosomatic conditions. Diaphragmatic breathing involves contraction of the diaphragm, expansion of the belly, and deepening of inhalation and exhalation, which consequently decreases the respiration frequency and maximizes the amount of blood gases. Benefits of diaphragmatic breathing have been investigated in association with meditation and ancient eastern religions (such as Buddhism) and martial arts (Lehrer et al., 2010). It is considered to be a core component of yoga and Tai Chi Chuan (TCC) and contributes to emotional balance and social adaptation (Sargunaraj et al., 1996; Beauchaine, 2001; Porges, 2001), as well as special rhythmic movements and positions.

Psychological studies have revealed breathing practice to be an effective non-pharmacological intervention for emotion enhancement (Stromberg et al., 2015), including a reduction in anxiety, depression, and stress (Brown and Gerbarg, 2005a,b; Anju et al., 2015). A 1-day breathing exercise was found to relieve the emotional exhaustion and depersonalization induced by job burnout (Salyers et al., 2011). A 30-session intervention with a daily duration of 5 min can significantly decrease the anxiety of pregnant women experiencing preterm labor (Chang et al., 2009). In addition, similar effects on anxiety was observed in a 3-days intervention study, where breathing practices were performed 3 times per day (Yu and Song, 2010). Further evidence from a randomized controlled trial (RCT) suggested that a 7-days intensive residential yoga program that included pranayama (breathing exercises) reduced anxiety and depression in patients with chronic low back pain (Tekur et al., 2012). Supportive evidence has also come from a line of RCTs of TCC and yoga (Benson, 1996; Telles et al., 2000; Oakley and Evans, 2014). Currently, breathing practice is widely applied in clinical treatments for mental conditions, such as post-traumatic stress disorder (PTSD) (Sahar et al., 2001; Descilo et al., 2010; Goldin and Gross, 2010), motion disorders (Russell et al., 2014), phobias (Friedman and Thayer, 1998), and other stress-related emotional disorders.

Earlier studies have observed an attention/vigilance impairment related to breathing dysfunction in dementia and sleep-disordered breathing in individuals across all ages (Chervin et al., 2006). More recent studies have suggested that there is a bidirectional association between breathing and attention. A growing number of clinical studies have demonstrated that breathing-including meditation may represent a new non-pharmacological approach for improving specific aspects of attention. Mindfulness, for instance, contributes to alerting and orienting, but conflicts with monitoring. In addition, an 8-weeks mindfulness-based stress reduction yielded a larger effect than a 1-month intensive mindfulness retreat, on the attention altering component (Jha et al., 2007). Focused attention meditation is a Buddhist practice, whereby selective attention and the sensation of respiration must be sustained (Gunaratana, 1993/2002; Gyatso and Jinpa, 1995). Three months of intensive focused attention meditation have been found to reduce variability in attentional processing of target tones and to enhance attentional task performance (Lutz et al., 2009). Some studies have investigated cognitive and emotional improvement simultaneously, and have indicated that a brief mental training could enhance sustained attention as well as reduce fatigue and anxiety (Zeidan et al., 2010). Some researchers believe that the relaxation generated by peaceful breathing helped to manage inattention symptoms among children with attention deficit-hyperactivity disorder (ADHD) (Amon and Campbell, 2008). These results led to the development of a breath-controlled biofeedback game called ChillFish, which improved children’s sustained attention and relaxation levels (Sonne and Jensen, 2016).

Studies orientated toward the physiological mechanism of breathing intervention effects have indicated a shared physiological basis underlying breathing, emotion, and cognition, involving the autonomic nervous system. Physiological evidence has indicated that even a single breathing practice significantly reduces blood pressure, increases heart rate variability (HRV) (Wang et al., 2010; Lehrer and Gevirtz, 2014; Wei et al., 2016) and oxygenation (Bernardi et al., 1998), enhances pulmonary function (Shaw et al., 2010), and improves cardiorespiratory fitness and respiratory muscle strength (Shaw et al., 2010). A daily 15-min breathing training for 2 weeks significantly promoted mean forced expiratory volume in 1 s and peak expiratory flow rate (Bernardi et al., 1998). Breathing with a certain frequency and amplitude was found to relieve clinical symptoms in patients of all ages with sleep-disordered breathing (Chervin et al., 2006). Evidence from yoga practice also confirms a reduction of sympathetic and an increase of parasympathetic nervous system activity (Vempati and Telles, 2002; Raghuraj and Telles, 2003). Cardiac vagal tone is assumed to form part of the shared physiological basis of breathing and emotion. It is influenced by breathing and is also integral to vagal nerve stimulation that is closely associated with the physiological basis of emotion, including emotional regulation, psychological adaptation (Sargunaraj et al., 1996; Beauchaine, 2001), emotional reactivity and expression, empathic responses, and attachment (Porges, 2001). Moreover, dysfunction of the autonomic nervous system is observed in adults with anxiety (Kawachi et al., 1995; Thayer et al., 1996; Friedman and Thayer, 1998), depression (Carney et al., 1995; Lehofer et al., 1997), PTSD (Sahar et al., 2001), panic disorder (Friedman and Thayer, 1998), and other stress-related mental and physical disorders (Benson, 1996; Becker, 2000; Bazhenova et al., 2001; Jacobs, 2001).

The shared physiological basis of attention and breathing can be detected in part in the autonomic nervous system of patients with ADHD (Beauchaine, 2001), but more evidence is provided by electroencephalographic (EEG) studies and functional magnetic resonance imaging (fMRI) studies (Lutz et al., 2004). For instance, EEG studies have suggested that regular breathing practice during yoga and meditation can increase β-activity in the left frontal, midline, and occipital brain regions (Bhatia et al., 2003; Snayder et al., 2006), which has been associated with enhanced cognitive performance, such as during attention, memory, and executive functions (Freeman et al., 1999). In addition, fMRI studies have also detected a significant increase in activation in the bilateral inferior frontal and temporal regions under meditation, as compared to a relaxation condition. Such studies implicated the right inferior frontal cortex/right insula and right middle/superior temporal cortex as the regions involved in meditation (Hernández et al., 2015).

Cortisol, a steroid hormone of the glucocorticoid class, is released in response to stress. Cortisol release is associated with depression, anxiety, and other negative emotions. The underlying mechanism may be grounded in its sensitivity for the activity of the hypothalamic–pituitary–adrenal (HPA) axis (Clow et al., 2010), which regulates metabolism, immunity, and some mental processing, including memories and emotional appraisal (Pariante and Lightman, 2008). Plasma cortisol levels reflect changes in the activation of the HPA axis with changes in CO_2_ inhalation (Argyropoulos et al., 2002), while salivary cortisol levels have been associated with fast withdrawal of attention in response to angry faces (van Honk et al., 1998). However, the associations between breathing, emotion, attention, and cortisol have not been tested together.

Although breathing practice offers an integrated benefit for mental and physical health, the results of studies on this topic are inconsistent, because of methodological limitations in the experimental design, a lack of measurable breathing feedback, and limited sample sizes. Most cross-sectional and longitudinal studies have focused on how breathing treatment benefits individuals with particular conditions, such as women during pregnancy (Schmidt et al., 2000; Booth et al., 2014) and clerks experiencing job burnout (Salyers et al., 2011), rather than on its health promotion function in a healthy population. Most importantly, most studies have investigated physiological effects, emotional benefits, and cognitive benefits separately, which prevents an understanding of the possible mental and physiological mechanisms of breathing in terms of its potential benefit for both mental and physical health.

The present study was a pilot RCT with visible feedback breathing recordings used to monitor the breathing performance overall and to evaluate the outcomes of breathing practice. The aims of this study were to investigate the mental benefits and the hormone levels in healthy volunteers who completed an 8-weeks breathing training scheme. An emotional self-reporting scale and cognitive tests were used to measure mental benefits. Additionally, cortisol a major HPA-axis-related stress hormone in humans (Matousek et al., 2010), was also measured to examine whether the breathing practice could be a buffer for modulating stress levels in the working population. We hypothesized that an 8-weeks breathing training course would significantly improve cognitive performance, and reduce negative affect (NA) and physiological stress.

## Materials and Methods

### Participants

Participants were recruited from a local IT company in Beijing, China. The Institute Review Board of Beijing Normal University approved this study. This study was performed in accordance with the ethical standards laid down in the 1964 Declaration of Helsinki and its later amendments. The procedure of the study was fully explained to the participants, and informed written consent was obtained from each participant before the study.

All participants completed the following screening forms: (1) a health approval from a recent physical check-up at a medical center and (2) a demographic questionnaire that included basic demographic information and mind–body training experience. Participants who had a history of physical health problems, such as cardiovascular or cerebrovascular diseases, respiratory diseases, autoimmune diseases, diabetes, neuropathy, and drug or alcohol abuse, were excluded from the study. In addition, participants who had yoga, TCC, or Qigong experience, as well as other mind–body training, were excluded.

### Experimental Protocol

All interventions and tests were performed in a sunny, soundproof, open-air conference room at the IT company rather than in our laboratory. We reasoned that this would avoid any potential anxiety that could be brought on by rushing from the workplace to the laboratory. Participants sat comfortably in leather armchairs throughout the study.

A final total of 40 participants were included in this study. They were assigned to a breathing intervention group (BIG, 10 females and 10 males) or a control group (CG, 10 females and 10 males) by alternating the order of their registration. Gender balance was also taken into consideration during this sampling procedure.

The BIG learnt basic knowledge and essential skills about diaphragmatic breathing, and became familiar with experiencing breathing in as deeply as possible and then exhaling almost all the air from the lungs, slowly, in a self-controlled, slow rhythm, under the guide of a coach. All participants were instructed to focus on their breathing and the sensations produced in the body, while sitting comfortably in chairs with their eyes closed. Participants were considered as performing diaphragmatic breathing if their respiratory rate decreased while their respiratory amplitude increased in waveform.

After this learning phase, both groups completed the baseline tests. These included the Positive and Negative Affect Schedule (PANAS), the Number Cancellation Test (NCT), and a cortisol test. Thereafter, the BIG received 20-sessions of breath-controlling intervention. Each intervention involved a 15-min resting breathing session and a 15-min diaphragmatic breathing session consequently. The diaphragmatic breathing session began with general verbal guidance from the breathing coach, who spoke at a slow speed to help participants to become more easily involved. A final test, similar to the baseline test, was implemented at the end of the 20th intervention. In contrast, the CG received only an introduction of breathing and rest, a baseline test, and a final test, without any other intervention.

**FIGURE 1:**
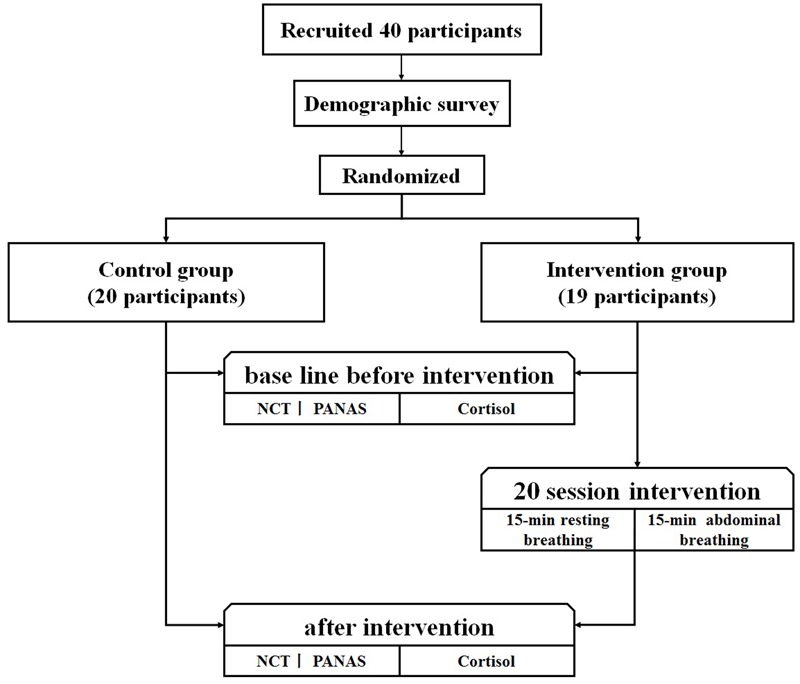
Experimental procedure of intervention.

The experimental training procedure consisted of 20 sessions over a period of 8 weeks. Each session was conducted every other day on weekdays. Both groups were informed of the purpose and the procedures of this study after training. In the BIG, 20 participants were required to conduct resting breathing for 15 min and diaphragmatic breathing for 15 min in each session (**Figure 1**). During resting breathing, participants were instructed to breathe in a normal state. With closed eyes while sitting comfortably. During diaphragmatic breathing, they were instructed to inhale as deeply as they could while their abdomen expanded, and to exhale as slowly as they could while their abdomen contracted, in a self-paced rhythm, under the instruction of a breathing training coach and with feedback via a recording device. The two breathing conditions were recorded during the entire 30-min training. Therefore, each participant’s breathing waves were visible and monitored by the experimenter to ensure that participants were following the instructions completely. For the CG, data of resting breathing and diaphragmatic breathing from 20 participants were collected on the first day of training and on the last day of training. Before and after the entire training, the two groups completed the PANAS, NCT, and cortisol level test (**Figure 1**).

### Breath Recording

Synchronous breathing signals were recorded using breathing monitors (Dongtuo Science and Technology Ltd., Beijing, China) that recorded participants’ respiration with a high temporal resolution. All data were collected by inductive sensors (JD/PW-5; Boda Electron Co., Beijing, China), which were kept against the chest of each participant during the rest condition, or alternatively against the abdomen during diaphragmatic breathing. Data were transferred to the host computer (Lenovo, M4600 P3.0HT 25640VN) via Bluetooth and expressed on-screen as continuous visual waveforms. The height of the wave crest represented the amplitude of a single breath, while the wavelength indicated the duration. Each computer was connected to two inductive sensors; 10 recordings were made simultaneously.

All five research assistants were postgraduate students in psychology. They attended a meeting in which training was given and breath recording was practiced for approximately 1 week prior to the formal experiment, in order to standardize the data collection procedure. Each of them monitored breathing processes for two participants concurrently and ensured that data input and output proceeded smoothly.

Throughout the breathing process, all participants kept their eyes closed and breathed through the nose. The assistants supervised their recordings and obtained the help of the coach if the recording vibrated or if the breathing frequency remained higher than 6 breaths/min by 2 min after the intervention began. The coach offered special guidance to help these participants attain diaphragmatic breathing.

### Psychological Measurements

All participants were asked to complete behavioral measurements before training and after training. The behavioral measurements included the PANAS and the NCT. The PANAS (Watson et al., 1988) is a 5-point Likert scale that measures participants’ feelings during the past week. It includes 20 self-reported items that are equally divided into a positive affect (PA) subscale and a NA subscale. Items are scored on a scale of 1–5, indicating very slightly or not at all, a little, moderately, quite a bit, and very much, respectively. The PANAS scale is highly internally consistent, largely uncorrelated, stable, and reliable across cultures, and can distinctively estimate PA and NA at the same time (Thompson, 2007).

The NCT includes one short practice sheet and four test sheets for evaluate attention sustainability, which is reflected by the scores generated according to the accuracy in the test. Each sheet contains 200 single digits with special symbols below or above them. The targets are “9” digits with two symbols below, above, or on either side. Participants were asked to cross out targets with a slash and ignore targets placed subsequent to a “5” as quickly as possible, within 1 min for each sheet. Participants had a 10- to 20-s interval break between two sheets. The final scores were yielded by the sum of the correct number of target digits for each sheet.

### Neuroendocrine Test

Salivary cortisol was collected four times with the Salivette^®^ Cortisol (Art. No. 51.1534) (Sarstedt AG and Co., Nümbrecht, Germany) at two time points: before and after diaphragmatic breathing at baseline, and before and after the diaphragmatic breathing at the final test. All salivary cortisol samples were processed according to the manufacturer’s instructions. Before collection, all participants were required to take a 5-min break. Each participant was asked to refrain from eating or drinking (except water) within 20 min before saliva collection. In both the BIG and CG, the saliva sample was collected between 11:00 and 12:00 to control for the variation in cortisol levels over the circadian rhythm.

Salivary cortisol was analyzed using a competitive enzyme immunoassay (ELISA, DiaMetra^®^, Perugia, Italy), which had a 3–10 g/mL limit of cortisol, for collections before lunch, according to specifications provided by the manufacturer DiaMetra^®^. An intra-assay coefficient variation (9.8%) and an inter-assay coefficient variation (15%) were calculated according to Jaedicke et al. (2012) (Validation and quality control of ELISAs for the use with human saliva samples) (Kirschbaum and Hellhammer, 1994; Ogbureke and Ogbureke, 2015).

## Results

### Demographic Characteristics

Demographic characteristics of all the participants in each group are summarized in **Table 1**. The paired *t*-tests showed that there were no between-group differences in terms of age [*t*(38) = 1.31; *p* = 0.56, Cohen’s *d* = 0.420], years of education [*t*(38) = 1.47; *p* = 0.56, Cohen’s *d* = 0.465], or work experience [*t*(38) = 1.05; *p* = 0.22, Cohen’s *d* = 0.331]. These results indicate that the BIG and CG were well balanced in age, years of education, and work experience.

**Table 1:** Demographic characteristics in the breathing intervention group (BIG) and control group (CG).

Characteristics	BIG (*n* = 19)	CG (*n* = 20)	*t*	*p*
Age (years)	30.16 ± 5.11	28.25 ± 3.91	1.31	0.56
Education (years)	16.21 ± 1.03	15.85 ± 0.37	1.47	0.10
Work experience (years)	7.24 ± 5.34	5.75 ± 3.31	1.05	0.22

### Respiratory Rate

Descriptive statistics showed the average respiratory rate to be 4 times/min in the diaphragmatic breathing condition (*M* ± *SD* = 3.45 ± 1.86) and 17 times/min in the resting breathing condition (*M* ± *SD* = 17.51 ± 5.02) (**Table 2**).

**Table 2:** Respiratory rate in resting breathing and diaphragmatic breathing.

Session	Resting breathing (*n* = 19)		Diaphragmatic breathing (*n* = 19)	
	Mean	Standard deviation	Mean	Standard deviation
1	14.82	2.95	9.95	3.09
2	16.92	4.00	8.30	2.04
3	17.46	3.59	6.51	4.73
4	16.60	2.44	4.91	1.60
5	16.04	2.71	4.07	1.08
6	16.01	4.04	3.95	1.06
7	17.80	4.40	3.48	1.41
8	16.22	4.68	3.81	1.13
9	17.06	4.62	3.32	1.00
10	16.86	3.91	3.19	1.30
11	19.73	9.95	2.80	1.13
12	18.12	5.83	2.72	0.60
13	15.81	4.44	4.32	1.49
14	18.00	3.28	4.01	1.63
15	16.84	5.35	2.88	0.96
16	16.89	3.58	3.46	1.08
17	17.34	4.32	4.30	1.46
18	17.59	4.88	3.89	1.24
19	17.43	3.77	3.46	0.97
20	17.51	5.02	3.45	1.67

A 2 × 20 within-subject repeated measures analysis was conducted to analyze the change in respiratory rate between the resting and diaphragmatic breathing conditions across all time-points, after each of the 20 sessions, in the BIG. The within-group factors were breathing conditions (diaphragmatic vs. resting) and intervention times (20 assessments). This analysis revealed a significant effect of time, *F*(19,133) = 2.09, *p* = 0.008, $\eta_{p}^{2}$ = 0.23, and a significant effect of breathing condition, *F*(1,7) = 99.60, *p* < 0.000, $\eta_{p}^{2}$ = 0.93. There was also a significant interaction between time and condition, *F*(19,133) = 5.28, *p* < 0.000, $\eta_{p}^{2}$ = 0.43. The simple effect revealed that there were significant frequency drops (compared to time point 1) at time point 8, *MD* = 2.36, *p* = 0.027, time point 11, *MD* = 1.77, *p* = 0.018, time point 13, *MD* = 2.38, *p* = 0.019, time point 15, *MD* = 2.53, *p* = 0.006, time point 16, *MD* = 2.28, *p* = 0.014, time point 18, *MD* = 1.61, *p* = 0.033, and time point 20, *MD* = 2.23, *p* = 0.023. The simple effects on breathing shows that the respiratory frequencies in diaphragmatic breathing were significantly below that in resting breathing at every intervention time point, with the MDs ranging from 5.63 to 15.93, all *p*s < 0.002. These results indicated that the breathing intervention successfully reduced the breathing frequency in the diaphragmatic breathing condition in the BIG.

**FIGURE 2:**
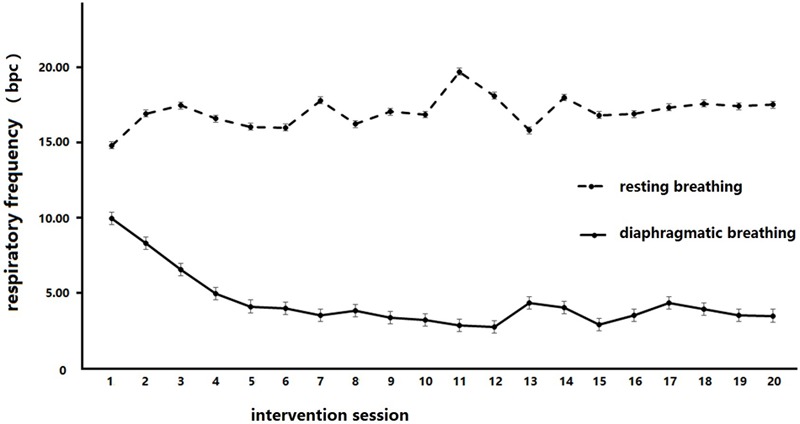
Respiratory frequency changes in breathing intervention group (BIG) under different breathing conditions. Diaphragmatic breathing frequency was significantly lower than that in the resting breathing condition in BIG.

A 2 × 2 mixed repeated measures analysis was conducted to analyze the change in the respiratory rate between the resting and diaphragmatic breathing conditions across time points and groups (see **Figure 2**). The within-group factor was the intervention session (baseline test and final line test), while the between factor was group (BIG vs. CG). The reduction of breathing frequency between diaphragmatic and resting conditions was employed as the measure. This analysis revealed a significant main effect of condition, *F*(1,36) = 23.36, *p* = 0.000, $\eta_{p}^{2}$ = 0.39, and an interaction between condition and groups, *F*(1,36) = 7.66, *p* = 0.009, $\eta_{p}^{2}$ = 0.175. A simple effect measurement was conducted and revealed that there was no significant between-group difference during resting breathing, *MD* = 0.43, *p* = 0.861, but diaphragmatic breathing frequency was significant lower than that during resting breathing, *MD* = 7.12, *p* = 0.000. The respiration frequency in diaphragmatic breathing was significantly less than that in resting breathing in the BIG, *MD* = 9.19, *p* = 0.000, but no similar result was detected in the CG, *MD* = 2.50, *p* = 0.153. These results indicated that the diaphragmatic breathing intervention was effective in both the BIG and the CG, but a significant breathing frequency decrease was only observed in the BIG.

### Positive and Negative Affect

**FIGURE 3:**
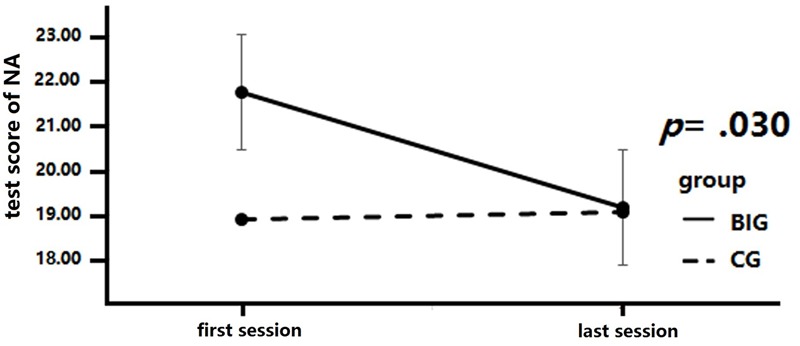
Negative affect (NA) score changes after intervention. Negative affect scores are significantly decreased in the breathing intervention group (BIG) compared to the control group (CG).

A 2 × 2 mixed repeated measures analysis was conducted to analyze the change in NA across the intervention (see **Figure 3**). The between-group factor was group (BIG vs. CG), while the within-group factor was test time (baseline test vs. final test). Time and group revealed a marginally significant interaction, *F*(1,37) = 3.43, *p* = 0.07, $\eta_{p}^{2}$ = 0.09. A simple effect measurement was conducted and revealed that the BIG demonstrated a significant reduction in NA score after the intervention, *MD* = 2.55, *p* = 0.02, while no similar results were detected in the CG, *MD* = -0.15, *p* = 0.88. There was no significant main effect of time, *F*(1,37) = 2.72, *p* = 0.11, $\eta_{p}^{2}$ = 0.07, or of group, *F*(1,37) = 0.9, *p* = 0.34, $\eta_{p}^{2}$ = 0.02.

A 2 × 2 mixed repeated measures analysis was conducted to analyze the change in PA across the intervention. We measured the between-group differences (BIG vs. CG) in PA at the baseline test and final test and detected an insignificant interaction between group and test times, *F*(1,37) = 0.17, *p* = 0.68, $\eta_{p}^{2}$ = 0.005, a non-significant main effect of time, *F*(1,37) = 0.96, *p* = 0.33, $\eta_{p}^{2}$ = 0.03, and a non-significant main effect of group, *F*(1,37) = 0.29, *p* = 0.60, $\eta_{p}^{2}$ = 0.008.

### Sustained Attention

**FIGURE 4:**
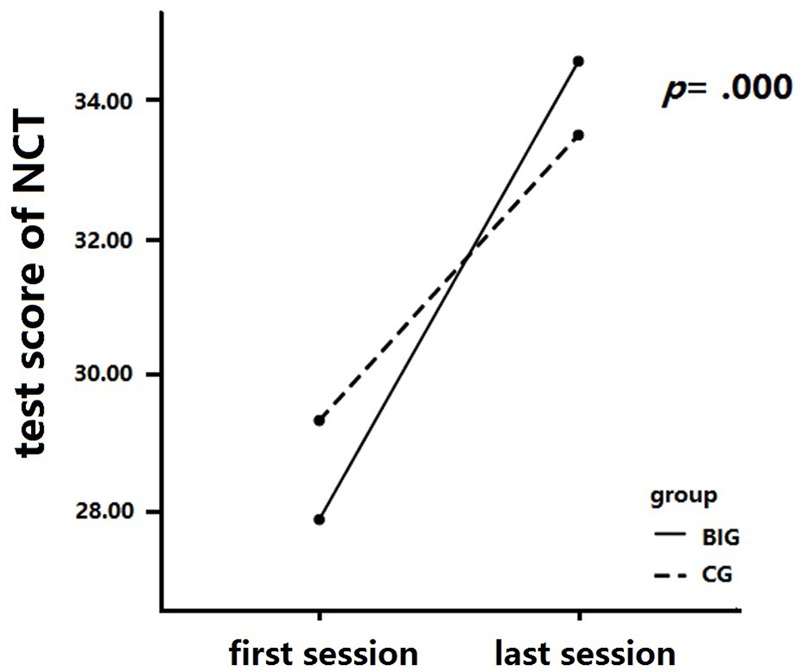
Number cancellation test (NCT) score changes after intervention. The BIG showed higher scores in sustained attention than did the CG after training, while there was no significant difference between these two groups after training.

A 2 × 2 mixed repeated measures analysis was conducted to analyze the change in the NCT score across the intervention (see **Figure 4**). We measured between-group differences (BIG vs. CG) in the NCT score change at the baseline and final tests. The NCT result revealed a significant interaction between time and group, *F*(1,37) = 9.68, *p* = 0.004, $\eta_{p}^{2}$ = 0.21. A simple effect measurement was conducted and revealed that the BIG showed a significant increase in the NCT score after the intervention, *MD* = 6.728, *p* = 0.000), and similar results were detected in the CG, *MD* = 4.19, *p* = 0.000. The main effect of time was significant, *F*(1,37) = 191.48, *p* = 0.00, $\eta_{p}^{2}$ = 0.84, so was the main effect of group, *F*(1,37) = 0.01, *p* = 0.93, $\eta_{p}^{2}$ = 0.00.

### Salivary Cortisol

**FIGURE 5:**
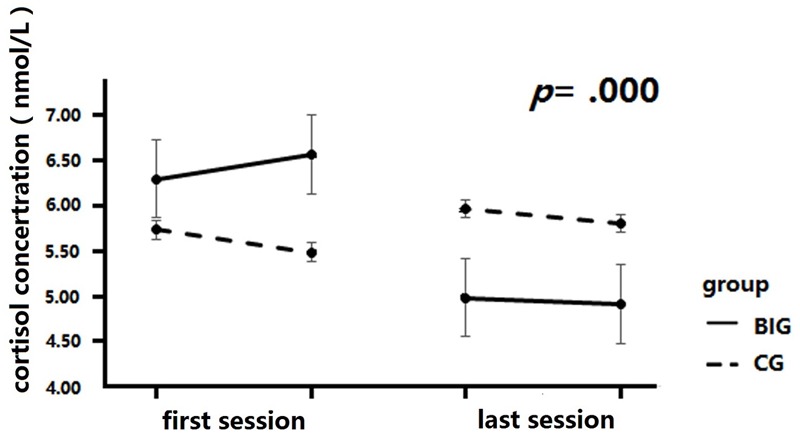
Cortisol concentration changes after intervention. Cortisol levels were significantly decreased after training in BIG, but no significant changes were detected in the CG.

A 2 × 4 mixed repeated measures analysis was conducted to analyze the change in salivary cortisol concentration across the intervention (see **Figure 5**). The between-group factor was group (BIG vs. CG), while the within-group factor was test time (test 1, test 2, test 3, and test 4). The salivary cortisol samples were collected before and after diaphragmatic breathing for both baseline and final tests. The concentration result revealed a significant interaction of time and group, *F*(3,111) = 9.06, *p* = 0.000, $\eta_{p}^{2}$ = 0.20. A simple effect measurement revealed that the BIG showed a significant decrease in salivary cortisol concentration after the intervention, whereby the concentration was significantly lower in test 3 and test 4 as compared to test 1 and test 2, *MD*_1-3_ = 1.32, *p* = 0.003, *MD*_1-4_ = 1.39, *p* = 0.002, *MD*_2,3_ = 1.59, *p* = 0.00, *MD*_1-3_ = 1.66, *p* = 0.00. However, no similar result was found in the CG, *p* > 0.05. The main effect of time was significant, *F*(1,37) = 4.17, *p* = 0.008, $\eta_{p}^{2}$ = 0.10, but there was no significant main effect of group, *F*(1,37) = 0.01, *p* = 0.92, $\eta_{p}^{2}$ = 0.00.

## Discussion

Using a randomized controlled design, the present study examined whether 8 weeks of intensive diaphragmatic breathing training, a core component of mind–body practices, could influence cognition, emotion, and physiological responses. As expected, the lowered frequency of respiration after the intervention suggested that the diaphragmatic breathing intervention had been inculcated. The NA score decreased after the intervention, but the PA did not change. Sustained attention scores increased after the intervention. Moreover, a significant time effect of diaphragmatic breathing on cortisol levels was observed (before training vs. after training tests), although no group differentially influence cortisol. We interpret the findings as illustrating the potential benefits of diaphragmatic breathing practice for improving cognitive function and reducing negative affect and physiological responses to stress in healthy adults.

### The Effectiveness of Breathing Practice

Numerous studies in health psychology and clinical treatment have demonstrated that diaphragmatic breathing is an effective relaxation technique in complementary and alternative medicine, with beneficial effects on physical and mental health (Cahalin et al., 2002; Tsang et al., 2015; Chen et al., 2016). However, in most studies of mind–body intervention, diaphragmatic breathing worked as a latent component or an essential preparation for the core intervention regime, such as meditation, TCC, or yoga (Telles et al., 2000; Oakley and Evans, 2014). In the present study, we monitored the breathing as an independent mind–body intervention form to discuss the health contribution to cognition, emotion, and stress response when respiration slowed. In order to achieve this aim, we adopted a unique breathing control method, which combined a monitoring device and coach supervision simultaneously.

In previous studies, practice duration and times were key characters for depicting an intervention protocol, but these seldom reported the final breathing rate and the manner in which respiration frequency decreased. In a study of slow-breathing training on chronic heart failure (Drozdz et al., 2016) reported its protocol as a 10–12 weeks’ (15 min per day) slow-breathing training with a breathing rate at 6 breaths/min, which meets the key requirements of diaphragmatic breathing. A clear respiration frequency provides a reliable and reproducible operation standard for estimating whether the intervention successfully decreased the respiration frequency. In the present study, the significant difference in respiration rate between the diaphragmatic breathing and resting breathing conditions confirmed that participants correctly followed the protocol for diaphragmatic breathing. The non-significant between-group difference in the respiration frequency in the resting breathing condition confirmed the distinction between the two breathing conditions. In addition, the continuous decrease in respiration rate observed in the BIG represented the process of learning and practice. All these results indicate that the change in respiratory rate could be attributed to the effectiveness of the diaphragmatic breathing practice over the 8 weeks. They also indirectly implied that the positive effects in this study were induced by intensive diaphragmatic breathing practice, rather than any confounding variables.

The present study employed a breathing monitor with results visualized on a screen and with a senior yoga coach providing professional guidance and instruction. Previous studies have demonstrated that most participants could follow self-paced breathing instructions via audio or video cues. Clinical studies usually adopt a breathing device with quantitative feedback parameters, such as respiratory rate or HRV (Sherlin et al., 2010). Respiratory sinus arrhythmia biofeedback has also been used to examine the effect of breathing practice on HRV and symptoms of PTSD, which revealed a significantly greater reduction in depressive symptoms and increases in HRV indices for individuals with respiratory sinus arrhythmia (Zucker et al., 2009). These breathing devices with feedback parameters have an advantage over visual or auditory instructions, without feedback (Brown et al., 2013), and have been widely applied in treatment for physical and mental disorders. Inviting a senior breathing coach is an alternative solution for breathing practice and control (Drozdz et al., 2016). In the present study, the coach strictly guided and monitored the entire process of diaphragmatic breathing during 20 sessions in a previous study. These two methods guaranteed that the participants breathed properly under two conditions under specific supervision during training. Having an appropriate monitor was another problem that was encountered in breathing practice studies.

### Breathing Practice to Decrease Negative Affect

Emotional improvements have been reported to be the most obvious benefit of mind–body interventions (Stromberg et al., 2015). It has been suggested that the detrimental effects of stress and negative emotions could be counteracted by different forms of breathing techniques, meditation, and relaxation (Jerath et al., 2015), as well as by yoga and TCC (Benson, 1996; Telles et al., 2000; Oakley and Evans, 2014). A non-randomized study suggested that a 1-week breathing practice decreased the mean of Post-traumatic Checklist-17 (PCL-17), Beck Depression Inventory (BDI-21), General Health Questionnaire (GHQ-12) in survivors of the 2004 South-East Asia tsunami. Their results indicated an equivalent effect of breathing practice with a traumatic incident reduction exposure therapy. Moreover, the effects persisted for at least 24 weeks after the intervention had finished (Descilo et al., 2010). As a non-pharmaceutical treatment, breathing control therapy is now wildly used in dealing with depression (Tsang et al., 2006), PTSD (Descilo et al., 2010), insomnia (Manjunath and Telles, 2005), and other relevant mental disorders (Brown and Gerbarg, 2005a). It is also applied as an adjuvant treatment for patients with physical disorders, including stroke (Marshall et al., 2014) and cancer (Hayama and Inoue, 2012). All these lines of evidence confirmed the efficacy of diaphragmatic breathing in clinical conditions, but we have shown its benefit for healthy individuals. A previous study has also reported that a better breathing technique was associated with greater reductions in anxiety (Sherlin et al., 2010). A 6-weeks’ breathing training course was long enough to cause a significant decrease in anxiety levels in healthy adults (Chandla et al., 2013). Evidence from diaphragmatic breathing studies suggested a significant reduction in the state anxiety after an 8-weeks’ intervention measured using the Beck Anxiety Inventory Assessment in adults (Chen et al., 2016), and a decrease in self-reported feelings of state anxiety and test performance in primary school students, by a pre-test/post-test, training-versus-control experiment (Khng, 2016). In the present study, our 20 sessions of diaphragmatic breathing practice significantly decreased the NA scores in the BIG. This is consistent with previous results and suggested a relief of basic NA in individuals’ daily lives. Although we did not detect the time point at which the emotional benefit occurred, previous studies suggested that even a one-time intervention could reduce stress, disengaged coping (Arsenio and Loria, 2014), and could provide certain curative alleviation of job burnout, as well as other emotional disorders. In that case, it remains unknown whether the reduction of NA occurs after the first intervention or after a significant reduction in respiration frequency. This could indicate that diaphragmatic breathing can provide an emotional improvement as a potential health care effect in healthy volunteers.

### Breathing Practice to Enhance Sustained Attention

Sustained attention is critical for maintaining performance over a period of time. Deficits in sustained attention are major symptoms for several mental disorders (Winterer et al., 2000). In normal healthy participants, fatigue, work burnout, and task difficulty usually lead to poor performance in sustained attention (Aston-Jones et al., 1999). In the present study, we investigated the diaphragmatic breathing as a single intervention method for sustained attention improvement. The results suggested that 20 session’s intervention provided an improvement in the NCT score. Indirect evidence supported these results, obtained from studies about breathing involving meditation (Lutz et al., 2009) and yoga training (Velikonja et al., 2010). In previous studies, both long-term intervention (MacLean et al., 2010), lasting for weeks, and short-term (Tang et al., 2007) intervention, for a few days, were effective. Consistent with previous studies, we have detected both long-term benefits after completing the intervention as a whole in the BIG and an immediate improvement in the CG. Notably, attention improvement was gained after 15 min of diaphragmatic breathing, which was markedly shorter than the 5 days’ training reported in a previous study (Tang et al., 2007).

Previous studies have hypothesized that perceptual improvements (MacLean et al., 2010) and stress reduction (Jensen et al., 2012) are the mechanisms by which attentional improvement is gained. Combining the increase in the NCT score and the decrease in the NA observed in the present study, we propose that relaxation gained from diaphragmatic breathing improved the attention test performance (Amon and Campbell, 2008; Sonne and Jensen, 2016). From the point of view of neuroscience, adjusting the imbalances in the autonomic nervous system is the unique contribution provided by breathing intervention, and was directly supported by TCC research. It indicated that the HRV increased when diaphragmatic breathing was performed, which indicated an activity balance between the sympathetic and parasympathetic systems (Wei et al., 2016). Therefore, it is reasonable to infer that diaphragmatic breathing might modulate cognitive performance by predominantly exerting its influence on the autonomic nervous system. Although the neuro-mechanism remains to be clarified, it is likely that deep breathing could link mind and body together to regulate the information processing related to attention.

### Breathing Practice and Physiological Responses

Cortisol is a reliable indicator of stress (Feder et al., 2009), because its concentration increases when having to cope with stressful events. We employed salivary cortisol as a measure of physiological response in the present study to estimate the neurophysiological benefit of diaphragmatic breathing. Its concentration decreased significantly after the 20 sessions’ intervention, which was consistent with previous results from parents of children and adolescents with diabetes type 1 (Tsiouli et al., 2014). This result was consistent with previous studies and indicated that breathing practice reduced the stress-related physiological response level in healthy volunteers.

Cortisol is also closely associated with the HPA axis (Clow et al., 2010), which can involuntarily control metabolism, immunity, and some mental processing, including memory and emotional appraisal (Pariante and Lightman, 2008), and can easily be affected by breathing (Argyropoulos et al., 2002). Its association with attention (van Honk et al., 1998), as well as breathing practice, cognitive processing, and emotional arousal is strong. It has been suggested that the sympatho-vagal stress response returns to an optimal balance at 4.5–5.5 bpm breathing in most adults (Lehrer et al., 2010), but no direct evidence has illuminated the potential mechanism of this physiological effect.

A hypothesis provided by Jerath et al. (2015) suggested that breathing stimulates vagal activation of GABA pathways from the prefrontal cortex and insula, to inhibit amygdala over-activity (Brown et al., 2013). A tuning function of the brain toward a parasympathetically driven mode and positive states was observed in the left insula and left orbitofrontal cortex with increasing yoga experience (Villemure et al., 2015). Evidence from brain imaging studies has supported this hypothesis. These results suggested that long and regular breathing-involving meditation practice significantly deactivated the limbic system (Kalyani et al., 2011) and rostral prefrontal cortex (Tomasino and Fabbro, 2016), and increased the activation of the right dorsolateral prefrontal cortex (DLPFC) while performing an attention-focused task. Moreover, specific changes, such as an increased cortical thickness (Wei et al., 2013), prefrontal-hippocampus functional connectivity (Tao et al., 2016), and a decreased regional homogeneity in the DLPFC (Wei et al., 2014), were also detected during the resting state among TCC senior practitioners. A structural change was proposed according to the autonomic nervous evidence, and was supported by an absence of age-related gray matter decline in senior yoga practitioners, as seen on structural MRI (Villemure et al., 2015). All these results suggested that the prefrontal cortex served as the predominant commander, supervising the activity from the limbic system, and modulating the activity of the autonomic nervous system.

### Limitations

In the present study, we employed healthy volunteers as our target population. In that case, we estimated the improvement in mood and cognition and the physiological response of stress, rather than clinical symptoms, such as anxiety, depression, attentional impairment, or other stress-related pathological symptoms. In comparison to a previous study, our results investigated the application of diaphragmatic breathing as a daily health care for health population. Some of the limitations of the present study should be acknowledged. First, we discussed the diaphragmatic breathing benefits from the points of view of emotion, cognition, and physiology, respectively, rather than by combining these three aspects together to obtain further mechanism-related results. Therefore, we have deduced a limited contribution to elucidating the underlying associations between cognitive progressing, affective improvement, and physiological change. Moreover, in terms of the neuroendocrine response, the cortisol levels were reduced after a 20 interventions, but no reference criteria were provided to demonstrate whether this decrease resulted in a real physiological benefit or was only statistically significant.

In future studies, it would be interesting to investigate the time-course between NA reduction and a sustained attention score increase. We expect that a time-course would elucidate the association between emotional and cognitive benefit, and allow testing of the hypothesis that the relaxation induced by diaphragmatic breathing relieves stress and thereby benefits cognitive possessing.

## Conclusion

The present study illustrates the potential for diaphragmatic breathing practice to improve cognitive performance and reduce negative subjective and physiological consequences of stress in healthy adults. Despite the promise of diaphragmatic breathing practice in supporting function and health, further investigation is needed to delineate mechanisms that underlie these benefits.

## Ethics Statement

The effect of diaphragmatic breathing exercise on attention, negative affect and stress level: a preliminary randomly controlled study on occupational population was approved by the ethical broad of school of psychology, Beijing Normal University. The method, experiment design, and safety of participants were strictly approved by the ethical broad of school of psychology, Beijing Normal University.

## Author Contributions

G-XW and Y-FL designed the work, drafted and finalized the manuscript. XM, Z-QY, HZ, N-YD, Y-TS, and Z-QG collected and analyzed the data, and revised the manuscript.

## Conflict of Interest Statement

The authors declare that the research was conducted in the absence of any commercial or financial relationships that could be construed as a potential conflict of interest.
